# Sterile inflammation of peritoneal membrane caused by peritoneal dialysis: focus on the communication between immune cells and peritoneal stroma

**DOI:** 10.3389/fimmu.2024.1387292

**Published:** 2024-05-08

**Authors:** Hongyong Su, Rong Zou, Jinqi Su, Xiaocui Chen, Haijuan Yang, Ning An, Chen Yang, Jixin Tang, Huafeng Liu, Cuiwei Yao

**Affiliations:** Guangdong Provincial Key Laboratory of Autophagy and Major Chronic Non-communicable Diseases, Key Laboratory of Prevention and Management of Chronic Kidney Disease of Zhanjiang City, Institute of Nephrology, Affiliated Hospital of Guangdong Medical University, Zhanjiang, Guangdong, China

**Keywords:** peritoneal dialysis fluids, non-bioincompatible, sterile inflammation, immune cells, mesothelial cells

## Abstract

Peritoneal dialysis is a widely used method for treating kidney failure. However, over time, the peritoneal structure and function can deteriorate, leading to the failure of this therapy. This deterioration is primarily caused by infectious and sterile inflammation. Sterile inflammation, which is inflammation without infection, is particularly concerning as it can be subtle and often goes unnoticed. The onset of sterile inflammation involves various pathological processes. Peritoneal cells detect signals that promote inflammation and release substances that attract immune cells from the bloodstream. These immune cells contribute to the initiation and escalation of the inflammatory response. The existing literature extensively covers the involvement of different cell types in the sterile inflammation, including mesothelial cells, fibroblasts, endothelial cells, and adipocytes, as well as immune cells such as macrophages, lymphocytes, and mast cells. These cells work together to promote the occurrence and progression of sterile inflammation, although the exact mechanisms are not fully understood. This review aims to provide a comprehensive overview of the signals from both stromal cells and components of immune system, as well as the reciprocal interactions between cellular components, during the initiation of sterile inflammation. By understanding the cellular and molecular mechanisms underlying sterile inflammation, we may potentially develop therapeutic interventions to counteract peritoneal membrane damage and restore normal function.

## 1 Introduction

Peritoneal dialysis (PD) is a widely used method for renal replacement therapy, similar to hemodialysis and renal transplantation. It involves using the peritoneal membrane as a dialysis membrane to treat end-stage renal disease (ERSD) (1–3). In this procedure, the peritoneal membrane cavity serves as a medium for the transfer of waste products and solutes between the body and the peritoneal dialysis fluids (PDFs). A permanent catheter is inserted to introduce PDFs into the peritoneal cavity. These PDFs contain glucose as an osmotic agent, which facilitates the movement of fluid from the circulation to the peritoneal membrane cavity, eliminating the metabolic waste products and excess water (4). Globally, PD is utilized by over 272,000 patients, accounting for approximately 11% of dialysis patients (5). PD therapy offers a higher quality of life and lower cost for individuals with ESRD compared to hemodialysis, owing to its simplicity of operation, low risk of cross-infectious, and preservation of residual renal function (6–8). Traditionally, the most commonly used PDFs have an acidic pH and rely on high osmotic glucose solutions to facilitate water and solute exchange. However, in recent years, various types of biocompatible PDFs, such as bicarbonate-based, icodextrin-based, amino acid-based solutions, have also become available in the market (9).

Although PD therapy greatly improves the quality of life for individuals with ESRD, it is not without its drawbacks. One notable concern is the occurrence of PD-related peritonitis, which can lead to chronic inflammation and damage to peritoneal cells (10–13). The development of peritonitis in PD can be attributed to both infectious and non-infectious factors (14). In particular, infection-related peritonitis remains the most common cause of technique failure and subsequent transition to hemodialysis in academic settings. The peritoneal membrane often undergoes significant changes in both structure and function during prolonged dialysis, leading to chronic inflammation. These alterations include mesothelial-to-mesenchymal transition, the growth of new blood vessels (neoangiogenesis), the development of fibrosis beneath the mesothelium (sub‐mesothelial fibrosis), and the occurrence of hyalinizing vasculopathy. These factors can cause irreversible damage to the peritoneal tissue, resulting in the failure of ultrafiltration and a decline in the effectiveness of dialysis (15–17). Unfortunately, the chronic sterile inflammation caused by these various factors frequently goes unnoticed or receives limited attention.

The induction of sterile inflammation is a complex pathological process in which resident cells sense pro-inflammatory signals and release extracellular mediators, leading to the recruitment of circulating immune cells that contribute to the initiation and escalation of the inflammatory response. Consequently, the development of sterile inflammation is the result of intricate signaling interactions between stromal resident cells and circulating immune cells, understanding of which has the potential to improve the medical management of this harmful condition. In recent years, there have been some successful attempts to address this issue. This paper focuses on the interactions between immune cells and peritoneal stroma cells in sterile inflammation. Furthermore, potential interventions for sterile inflammation caused by PD are discussed, which could be significantly important in preventing aseptic changes in PD.

## 2 Interactions between stromal resident cells and immune cells

### 2.1 Stromal Components of the Peritoneal Membrane: the Source of Inflammation

#### 2.1.1 Peritoneal mesothelial cells and fibroblasts: center of inflammation onset

The peritoneum is structured into three distinct layers: the mesothelium, the basal lamina, and the submesothelial stroma. The submesothelial stroma provides support to the mesothelial cells and the basal lamina, and is mainly composed of collagen fibers, (myo)fibroblasts, adipocytes, as well as lymphatic and blood vessels (18). In the context of PD, the prolonged exposure of the mesothelium to bioincompatible PDFs and their breakdown products triggers an inflammatory response in mesothelial cell. Consequently, this response stimulates the production of cytokines, chemokines, and extracellular matrix (ECM) proteins (19–23). These factors have the potential to initiate damage to the peritoneum (24). The occurrence of cellular stress and tissue damage triggers the production of ECM degradation products and the release of endogenous cellular constituents, which are referred to as damage-associated molecular patterns (DAMPs) (25). These DAMPs activate pro-inflammatory and pro-fibrotic signaling pathways. Toll-like receptors (TLRs), specifically TLR2 and TLR4, as well as the receptor of advanced glycation end products (RAGEs), plays a crucial role in recognizing and responding to DAMPs (25–28). They recognize a wide range of DAMPs that are released during tissue injury, including hyaluronan and fibronectin resulting from matrix degradation, as well as heat shock proteins and high mobility group box-1 (HMGB1) released due to cellular stress (25, 29–33). Activation of TLRs triggers the production of inflammatory and fibrotic cytokines, such as tumor necrosis factor-α (TNF-α), interleukin 6 (IL-6), IL-8 and transforming growth factor β1 (TGF-β1) (34, 35). However, the use of soluble TLR2 (a TLR inhibitor) has been found to reduce the development of sterile peritoneal inflammation and fibrosis (28, 36). Additionally, when mesothelial cells are exposed to PDFs, they release HMGB1 from the nucleus. This HMGB1 acts on the mesothelial cells in an autocrine manner, stimulating the expression of IL-8 and monocyte chemoattractant protein-1 (MCP-1) through the mitogen-activated protein kinase (MAPK) signaling pathways (36–38). Importantly, elevated levels of HMGB1 in the serum have been associated with microinflammatory conditions in continuous ambulatory peritoneal dialysis (CAPD) patients. Inhibition of HMGB1 has shown a protective effect on peritoneal function in peritonitis models (39, 40).

Mitochondrial dysfunction plays a role in the inflammatory response of human peritoneal mesothelial cells (HPMCs) derived from PDFs (41). A key function of mitochondria is the production of reactive oxygen species (ROS), which are involved in oxidative stress (42). Recent research has shown that mitochondria also release DAMPs, which play a significant role in inflammatory and immune responses (43). In addition, impaired mitochondria release DAMPs, such as mitochondrial ROS and mitochondrial DNA, which are recognized by the immune system and trigger an immune response (44). This response can further damage mitochondrial, leading to a cycle of mitochondrial dysfunction and activation of inflammation. Specifically, mitochondrial DNA has been shown to activate of TLRs, leading to an inflammatory response (45). Additionally, mitochondrial DNA can activate the NACHT, LRR and PYD domains-containing protein 3 (NLRP3) inflammasome, resulting in the secretion of pro-inflammatory cytokines IL-1β and IL-18 (46–49). High glucose levels in PDFs also contribute to mitochondrial damage and apoptosis in HPMCs (50, 51). Moreover, mitochondrial dysfunction in HPMCs is implicated in T cells differentiation, further exacerbating the inflammatory response (52). The inhibition of ROS could have a significant impact on the activation of NLRP3, production of ROS, and expression of IL-1β (53). Furthermore, the application of resveratrol has been shown to induce mitophagy/autophagy through adenosine monophosphate-activated protein kinase, resulting in a decrease in the inflammatory response by suppressing the NLRP3 inflammasome (49). Additionally, recent research has shown that paricalcitol can alleviate the epithelial-to-mesenchymal transition (EMT) of HPMCs triggered by TGF-β1 through the inhibition of the NLRP3 inflammasome and oxidative stress (54).

Numerous studies have provided evidence that nuclear factor-κB (NF-κB) pathway plays a crucial role in the PD-related sterile inflammation. In patients undergoing CAPD, the presence of various compounds and glycated proteins strongly activates the NF-κB pathway in HPMCs, leading to the release of inflammatory mediators such as IL-1β, IL-6 and TNF-α, as well as the activation of cyclooxygenase-2 (55). In rat models, NF-κB signaling pathways are also activated by high glucose and hypertonic PDFs through a protein kinase C (PKC)-dependent mechanism. This activation results in an increased synthesis of MCP-1 (22, 56). Additionally, the EMT process observed in PD patients, which is a common disorder is also influenced by the activation of NF-κB (20, 23). A study has shown that p38 plays a role in maintaining the expression of E-cadherin by suppressing TGF-β-activated kinase 1 (TAK1) NF-κB pathway, thereby inhibiting EMT in primary HPMCs (57). The activation of p38 may also lead to an increase in IL-8 transcription through NF-κB and post-transcriptional molecular mechanisms (58–60). Another study has indicated that the process of EMT is regulated by the ERK/NF-κB/Snail1 pathway in primary mesothelial cells from PD patients (20). However, the administration of parthenolide, a NF-κB inhibitor, has been shown to reduce inflammation and peritoneal fibrosis (PF) through the NF-κB/TGF-β/Smad pathway, thereby reducing the level of IL-6, TNF-α, and MCP-1 (61).

**Figure 1:**
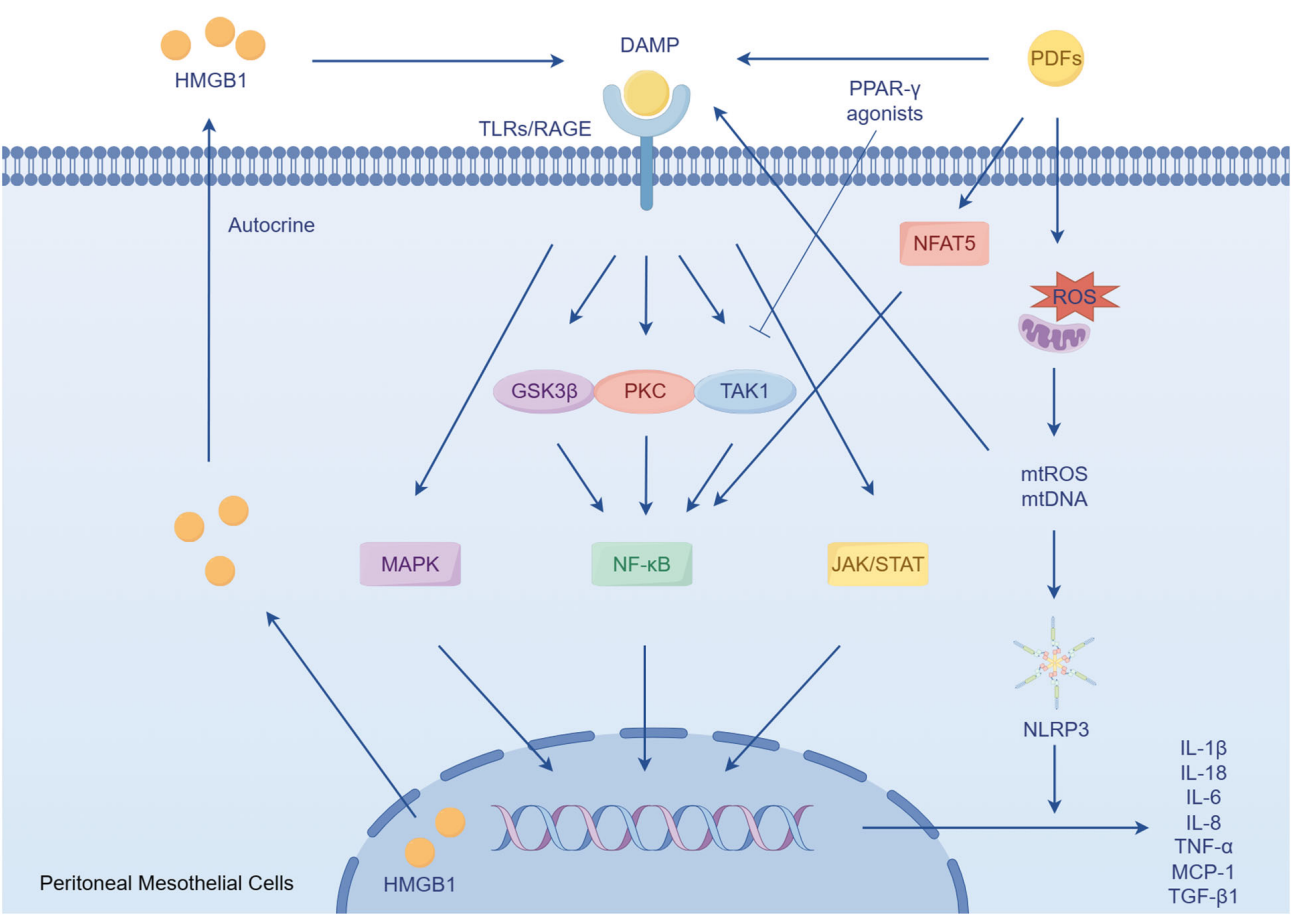
The role of peritoneal mesothelial cells in sterile inflammation. DAMPs accumulate during chronic exposure to bioincompatible PDFs. These DAMPs subsequently activate pro-inflammatory and profibrotic responses by activating TLRs and RAGE. In the condition of PDFs, the mesothelial cells secrets HMGB1, a type of DAMPs, which in turn enhances the inflammatory response. Furthermore, mitochondrial dysfunction induced by PDFs leads to the activation of NLRP3, triggering the secretion of the pro-inflammatory cytokines.

Recent researches have demonstrated that peroxisome proliferator-activated receptor-gamma (PPAR-γ), a nuclear hormone receptor that regulates glucose and lipid homeostasis, possesses anti-inflammatory properties (62, 63). Exposure of the peritoneum to PDFs leads to EMT of HPMCs, fibrosis, angiogenesis, and an inflammatory response. However, administration of the PPAR-γ agonist rosiglitazone in mice has been found to alleviate these changes (64). The PPARβ/δ agonist GW501516 has also been found to have a mitigating effect on peritonitis in rat models of PF through the inhibition of the TAK1–NF-κB pathway (65). Furthermore, microsomal prostaglandin E synthase-1 and its derived prostaglandin E2 also play a crucial role in PF (66). Recent researches have shown that the treatment of ONO-AE3–208, a prostaglandin E2 receptor 4, can suppress both the activation of the NLRP3 inflammasome and the expression of inflammatory cytokines induced by high glucose in rat peritoneal mesothelial cells. This is achieved by regulating NF-κB signaling (36, 67, 68). Additionally, the janus kinase/signal transducer and activator of transcription (JAK/STAT) signaling pathway has been found to contribute to the inflammatory response in the peritoneum of patients undergoing PD (69). Furthermore, both the p38 MAPK pathway and the PKC signaling have been shown to be activated in high glucose-induced inflammation in HPMCs (70, 71). It has also been observed that high glucose treatment stimulates IL-6 synthesis in Met-5A cell, and IL-6 subsequently stimulates vascular endothelial growth factor (VEGF) synthesis, partially dependent on the JAK/STAT3 signaling pathway (**Figure 1**) (72).

Mesothelial cells possess the remarkable ability to synthesize and release a variety of complement factors, specifically C4, C3, and C5-C9 (73, 74). These complement proteins play a crucial role in the immune response. Additionally, other tissue-resident cells, including immune cells, may also contribute to the production of complement proteins within the peritoneal cavity. Studies have confirmed the presence of complement system molecules, such as C3-C9 and factor D, in the fluid that is drained during PD (75, 76). In fact, proteomic analysis has identified up to 18 different complement proteins, including C3, C4, C9, factors D, B, H, and I, in the PDFs (77–81). It is noteworthy that the complement system can be activated within the peritoneal cavity due to the production and expression of various effectors (such as C3, C4, C5, and C6–C9) and their regulators (such as membrane cofactor protein, CD55, and CD59) by mesothelial cells derived from healthy individuals and patients with kidney disease who are undergoing PD (73, 74, 82, 83). Activation of the complement system in PD patients is believed to occur due to exposure to PDFs, particularly those containing high levels of glucose and glucose degradation products. This exposure to high glucose PDFs can trigger an excessive activation of the complement system, leading to local inflammation, cellular damage, and ultimately causing structural alterations in the peritoneal membrane. These alterations may include vascular proliferation, vasculopathy and PF (84, 85).

Glucose and its degradation products found in PDFs have been identified as triggers of EMT in mesothelial cells. This transition causes the cells to transform into fibroblast-like cells with increased migratory, invasive, and fibrogenic properties (37, 86). As a result, mesothelial cells undergoing EMT secrete large amounts of TGF-β and VEGF. The local production of VEGF by transitioning mesothelial cells appears to play a pivotal role in the mechanisms involved in peritoneal angiogenesis and vascular permeability. This process may contribute to the inflammation within the peritoneum (87, 88).

The EMT of mesothelial cells is a critical source of peritoneal fibroblasts, which have been implicated in the aseptic inflammation induced by PD. Similar to mesothelium cells, peritoneal fibroblasts, have the ability to synthesize various chemokines. One example is the release of chemokines MCP-1/CCL2 and IL-8/CXCL8, which are chemokines involved in recruiting immune cells, by peritoneal fibroblasts. This process is mediated by the NF-κB family, a group of transcription factors that play a role in inflammation (88). Additionally, peritoneal fibroblasts can produce cytokines of CXCL1 and CXCL8, which target neutrophils. The production of these cytokines is primarily induced by IL-1β (89). Furthermore, peritoneal fibroblasts have the capability to produce CCL5, a potent chemoattractant for mononuclear leukocytes. The production of CCL5 is intricately regulated by IFN-γ, a cytokine with immunomodulatory functions. Additionally, the treatment of peritoneal fibroblasts with IFN-γ can induce the production of CCL5 in response to CD40 ligand, a protein involved in immune cell activation (90).

Furthermore, peritoneal fibroblasts have been found to produce chemokines in response to exposure to high levels of glucose. *In vitro* studies have demonstrated that stimulation of peritoneal fibroblasts with high glucose PDFs leads to increased expression of chemokine (C-C motif) ligand mRNA and nuclear factor of activated T cells 5 (NFAT5) (91). Previous research has indicated that NFAT5 modulates the activity of NF-κB in response to osmotic stress (92). The increased expression of NFAT5 in human peritoneal fibroblasts is associated with NF-κB activation in patients undergoing PD, potentially leading to the recruitment of macrophages.

#### 2.1.2 Endothelial cells: the window of inflammatory cells infiltration

The presence of PD appears to have a notable proinflammatory effect on the endothelium (93). In healthy individuals, the expression of IL-17, a pro-inflammatory cytokine, is minimal in the peritoneum, but it is highly expressed in peritoneal biopsies of long-term PD patients (94). This increase in IL-17 contributes to angiogenic stimulation and direct damage to the peritoneum in PD patients. IL-17 up-regulates the expression of VEGF, leading to enhanced angiogenesis. It also promotes the production of CXC chemokines such as CXCL1 and CXCL8, which further contribute to inflammation.

**Figure 2:**
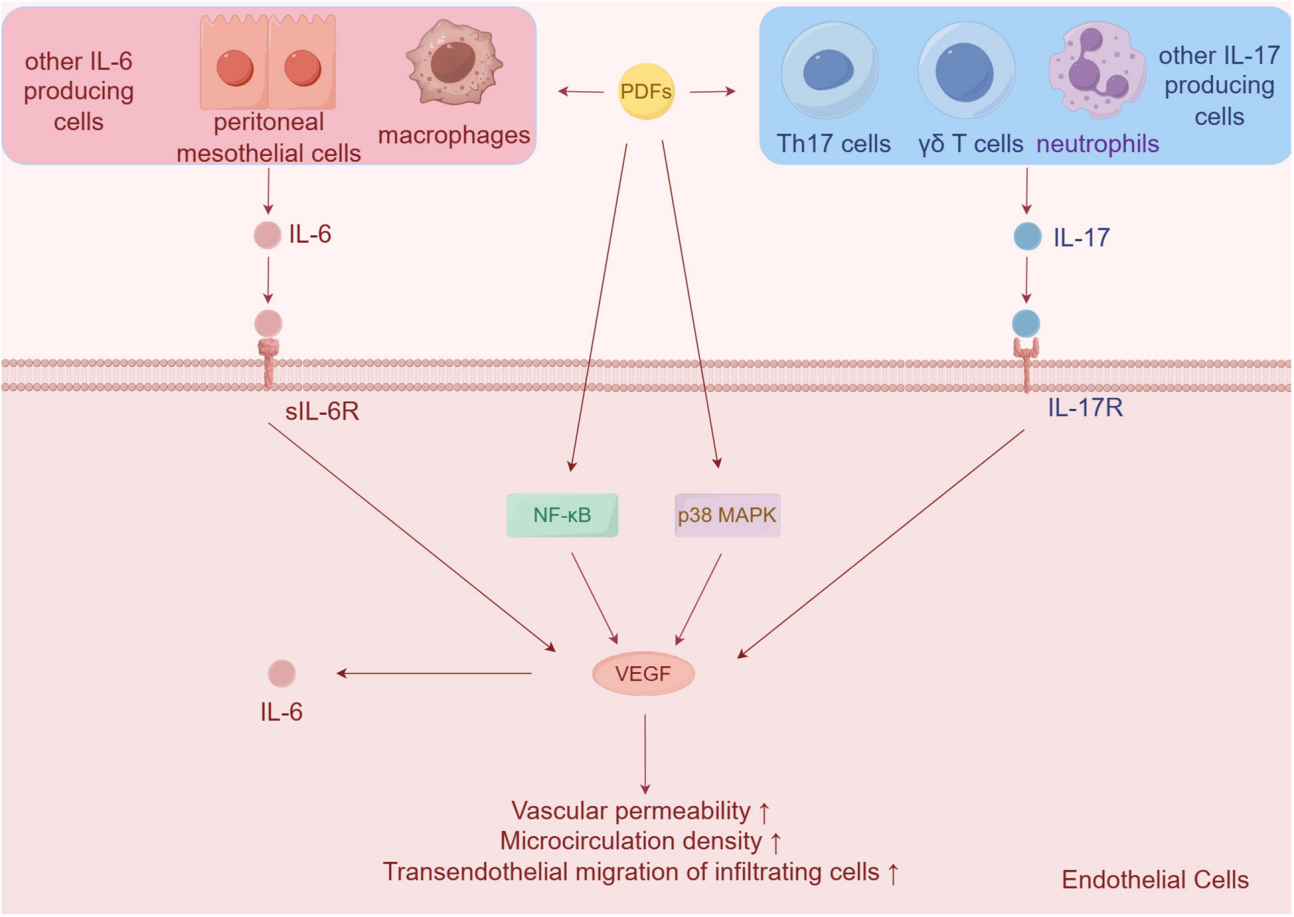
Endothelial cells alterations in PD. Both inflammatory cytokines IL-6 and IL-17 secreted from HPMCs and immune cells contribute to increase vascular permeability, microcirculation density, and transendothelial migration of infiltrating cells via up-regulating the expression of VEGF.

Additionally, IL-17 stimulates the release of IL-6, another pro-inflammatory cytokine, from various sources including HPMCs, endothelial cells, macrophages, and monocytes (95, 96). IL-6 upon, specific binding to its receptor, further enhances the production of angiogenic molecules like VEGF and adhesion molecules such as intercellular adhesion molecule 1 (97–99). With the stimulation of PDFs, the above inflammation factors were significantly higher in the endothelial cells via activating both p38 MAPK and NF-κB pathway. This observation was consistent with the findings in PD patients, where increased levels of these inflammatory factors were observed (93). The stimulation of endothelial cells by these inflammatory factors, such as VEGF, resulted in microvascular alterations characterized by increased vascular permeability, microcirculation density, and transendothelial migration of infiltrating cells. These alterations can contribute to endothelial cells proliferation, inflammation, and injury, ultimately leading to peritoneal inflammation, fibrosis, and reduced efficacy of PD (**Figure 2**) (100–102).

Previous studies have shown that cultured HPMCs produce VEGF-A and VEGF-C in response to glucose degradation products or cytokines (103, 104). However, it has been observed that targeting of VEGF-A by miR-15a-5p can suppress inflammation and fibrosis in HPMCs induced by PD (104). In addition, the use of biocompatible PDFs has been found to result in fewer adverse reactions in endothelial dysfunction compared to conventional PDFs in PD patients (105).

#### 2.1.3 Adipocytes: the neglected and important one

**Figure 3:**
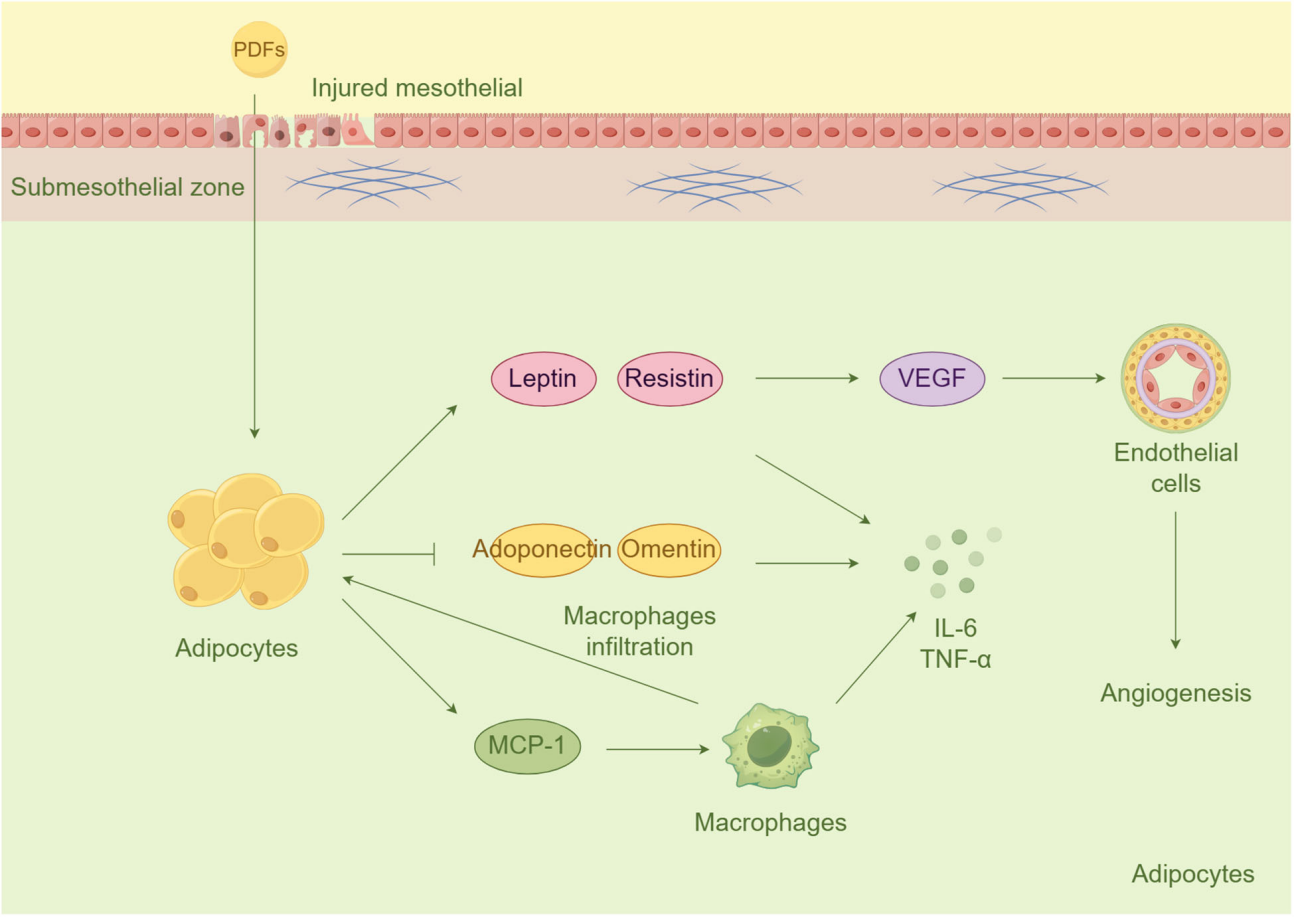
Adipocytes in inflammation and angiogenesis during PD. Adipocytes would be stimulated in the condition of injury mesothelial. The activated adipocytes subsequently release pro-inflammatory and pro-angiogenic cytokines (e.g., leptin and resistin) and suppress anti-inflammatory cytokines (e.g., adiponectin and omentin). Additionally, MCP-1 derived from adipose promotes macrophages infiltration to fat tissue.

In recent years, the potential impact of adipocytes in PD has been overlooked. There is a noticeable increase in body mass among PD patients, with visceral fat being the main contributor (106, 107). Long-term PD patients also display elevated levels of adipokines in their plasma (108). Ultrastructural investigations have shown that dialysate can penetrate adipose tissue in the presence of an injured mesothelial monolayer (109). Adipocytes have various functions, including autocrine, paracrine, and endocrine activities. They release a range of adipokines and cytokines, such as leptin, adiponectin, resistin, visfatin, IL-6, TNF-a, TGF-β, VEGF, and others (110). On one hand, adipokines such as leptin and resistin have pro-inflammatory and pro-angiogenic effects. Leptin, a hormone secreted in large amounts by adipocytes, can phosphorylate VEGFR2 and activate the p38 MAPK/Akt/COX-2 signaling pathway, thereby promoting angiogenesis (111). In addition, hyperleptinemia induced by PD also stimulates macrophages and monocytes to secret IL-6 and TNF-α (112). Conversely, certain adipokines, such as adiponectin and omentin, have anti-inflammatory and anti-angiogenic properties. However, their expression is suppressed in the peritoneum (113). Adiponectin plays a crucial role in inhibiting the production of adhesion molecules by endothelial cells, thereby preventing the attachment of monocytes (114). Moreover, it reduces the activation of NF-κB, which is induced by TNF-α. However, there is a negative feedback loop between TNF-α and adiponectin, as TNF-α downregulates the production of adiponectin, creating a vicious cycle that leads to lower adiponectin release (115). Furthermore, adipose-derived cytokine, promotes the propagation of inflammation by stimulation macrophage infiltration into the interstitial spaces within adipose tissue (116). Prolonged dialysis has been found to result in an accumulation of adipocytes and infiltrated macrophages within the body, which in turn secrete various adipokines and cytokines that contribute to inflammation and tissue damage (117–119). Interestingly, the omentum, a fatty tissue layer within the abdomen, contains a significant number of progenitor cells (120). The stromal vascular fraction derived from adipose tissues also contains pluripotent mesenchymal stem cells that have the ability to regenerate damaged tissue (121). This discovery offers a promising approach for preserving the functionality of the peritoneal membrane (**Figure 3**).

### 2.2 Immune cells: the complex signaling network

#### 2.2.1 Macrophages: master regulators of inflammation and fibrosis

**Figure 4:**
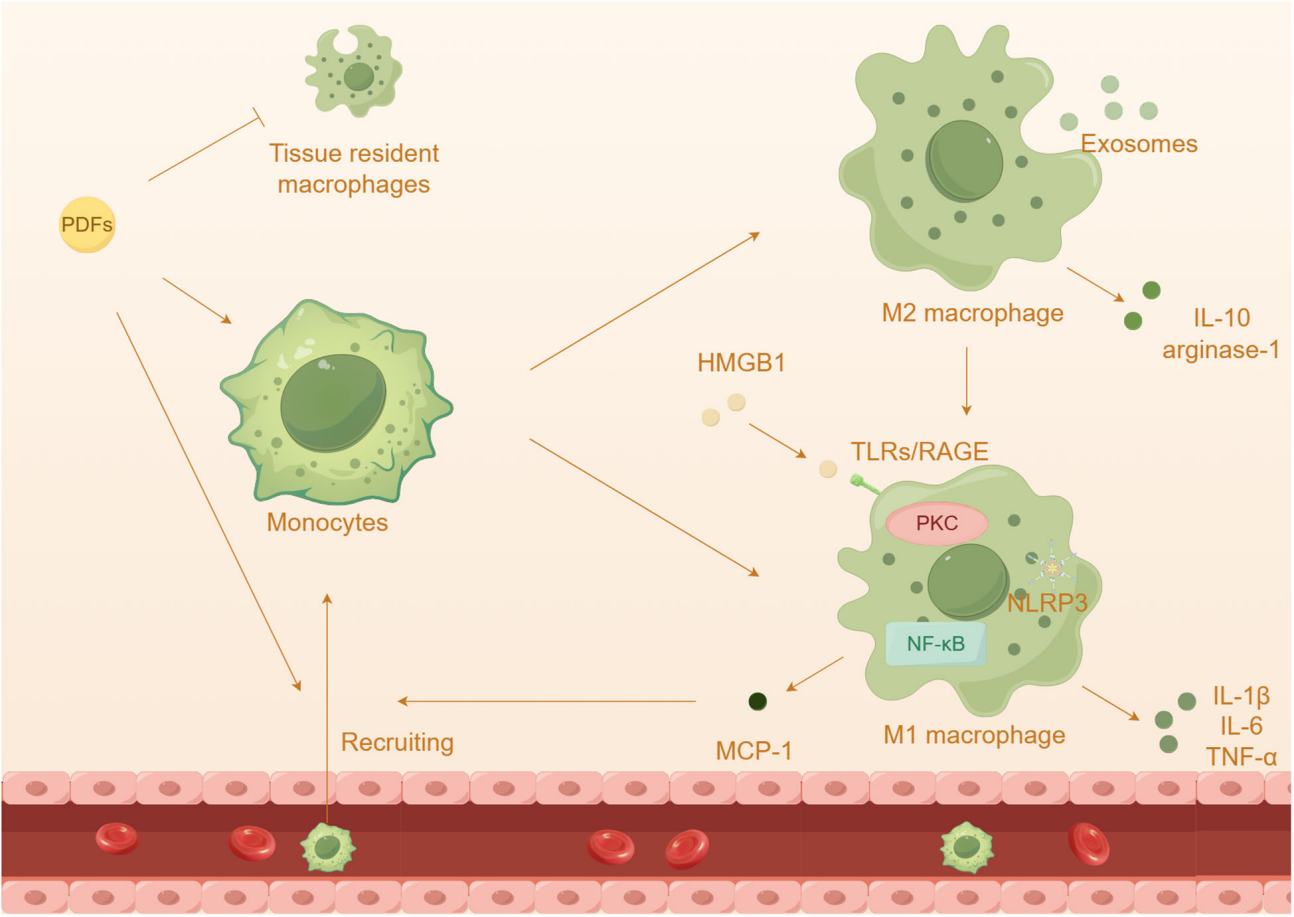
The effect of macrophages in peritoneal damage by PDFs. Prolonged dialysis further leads to an increased capacity of recruited macrophages and a depletion of tissue resident cells. Prolonged exposure to high glucose PDFs and the cytokines leads to the differentiation and activation of macrophages, which onsets inflammatory response.

The activation of macrophages plays a crucial role in the advancement of kidney diseases and the ineffectiveness of treatment for PD (**Figure 4**). Monocytes and macrophages constitute a significant proportion (50%-90%) of the infiltrated leukocytes in peritoneum (122, 123). Generally, macrophages can be divided into two categories: tissue resident macrophages and monocyte-derived macrophages (124). While monocyte-derived macrophages have been shown to contribute to the progression of inflammation and fibrosis, tissue resident macrophages have a protective role. Surprisingly, researchers have discovered that peritoneal resident macrophages are gradually losing their homeostatic properties and anti-inflammatory properties, instead exhibiting a heightened inflammatory response (125). Furthermore, it has been observed that M2 macrophages are elevated in both the effluents and peritoneal membrane biopsies of PD patients (126, 127). In mice induced with chlorhexidine gluconate and PDFs, there is also an increase in the infiltration of T cells and macrophages (128, 129). Additionally, the presence of high glucose PDFs leads to a significant increase in inflammation and almost complete depletion of tissue resident cells (125). The inflammatory macrophages that infiltrate the affected area produce inflammatory cytokines and collaborate with adipocytes, resulting in a substantial escalation of the inflammatory cascade (116).

Among these receptors, TLR4 has been extensively studied in macrophage polarization and peritonitis (59, 130). The injury and necrosis of mesothelial cells induced by high glucose exacerbate the accumulation of extracellular TLR4 and HMGB1, resulting in the recruitment of a significant number of macrophages to the abdominal cavity (131, 132). Additionally, HMGB1 has the ability to activate TLRs, initiating innate immunity and subsequently increasing the expression of MCP-1 and the activation of macrophages (133). Advanced glycation end products (AGEs) and glucose degradation products can stimulate peritoneal macrophages, leading to the secretion of cytokines such as IL-1β, IL-6, and IL-8, in HPMCs (134). The NLRP3 inflammasome and its downstream pathway enhances the activation of caspase-1 and the maturation of IL-1β specifically in macrophages, contributing to the progression of inflammation (135, 136). Prolonged exposure to high glucose PDFs and the cytokines can induce macrophage polarizations, which contributes to an inflammatory response and PF (137–139). M1 macrophages release inflammatory cytokines such as IL-1β, IL-6, and TNF-α, while M2 macrophages secrete anti-inflammatory substances like IL-10 and arginase-1 (140, 141). Previous research has shown that high glucose induces peritoneal injury via the PKC-β pathway and promotes M1 macrophage polarization in mouse models (142). It has been observed that PD induces a shift in adipose tissue macrophages from M2 to M1, promoting a pro-inflammatory state (116). Co-culturing HMrSV5 with M1 macrophages resulted in a loss of the typical epithelial cell morphology, indicating that HMrSV5 undergoes EMT through TLR4 receptors (143). Additionally, previous studies have proposed a role of M2 macrophages in peritoneal inflammation and PF (126, 144). It is worth mentioning that exposure to PDFs has been shown to induce polarization of M2 macrophages and the subsequent inflammatory response, which may be associated with the transmission of exosomes (145).

In previous research, it has been demonstrated that the administration of the probiotic *Lactobacillus casei Zhang* effectively corrects gut dysbiosis, leading to an improvement in PF. This improvement is achieved through the inhibition of macrophage-related inflammation via the PPAR-γ/NF-κB pathway (122). Moreover, the use of biocompatible PDFs has been found to increase the recruitment of M1 macrophages in uremic mice (146). In the context of dialysis-induced PF, mesenchymal stem cells have been shown to induce the polarization of macrophages into the M2 phenotype through the release of IL-6 (147). *Astragalus membranaceus*, a traditional Chinese medicine with anti-inflammatory properties, has been found to inhibit the recruitment and activation of monocytes/macrophages via the MCP-1 pathway in rats undergoing PD (148). Moreover, treatment with hepatocyte growth factor has been shown to reduce the infiltration of macrophages in mouse models of PF, while also mitigating the upregulation of proinflammatory and profibrotic genes associated with PF (149).

#### 2.2.2 Lymphocytes: the overactivated infiltrating immune cells

Lymphocytes can be roughly divided into T lymphocytes and B lymphocytes. B lymphocyte cells have been identified as important contributors to innate immunity and autoimmunity. However, there is little evidence suggesting their involvement in PD-related sterile inflammation (150). On the other hand, an increased number of T lymphocytes has been observed in the effluent of patients undergoing PD (151). Thus, targeting the differentiation of T cells may offer potential therapeutic strategies for treating peritoneal damage by regulating immune and inflammatory responses. Notably, there are notable differences in the levels of CD4+ and CD8+ T cells in patients undergoing PD compared to healthy individuals (152, 153). Regarding CD4+ cells subsets, Th1 cells are characterized by increased production of IFN-γ, while Th2 cells primarily secrete IL-4 (154). Interestingly, patients undergoing CAPD have shown a significant increase in the proportion of Th2 cells (155). This dysregulation of Th1/Th2 balance in PD patients leads to changes in proinflammatory and anti-inflammatory cytokines in their serum (156–158). Surprisingly, preliminary studies have indicated that PF mice induced by PDFs exhibit an enhanced immune response, characterized by the presence of Th17 and T cells in the peritoneum, rather than Th1 or Th2 cells (159).

Regulatory T cells (Tregs) play a crucial role in limiting inflammation, while Th17 cells secrete various proinflammatory cytokines. Tregs are responsible for regulating the expansion of T cells, including Th17 cells, which have been implicated in peritoneal damage and the development of fibrosis (151, 160). A primary study has demonstrated that exposure to PDFs leads to an imbalance of Th17/Treg cells, with an increase in Th17 cells and a decrease in Treg cells, ultimately resulting in peritoneal damage in mouse models (151). Additionally, CD69, a leukocyte membrane glycoprotein, contributes to activation of Th17 cells and the expression of IL-17 in PD mouse models through the JAK3/STAT5 signaling pathway (160, 161). Therapeutic inhibition of TLR2 activity in the peritoneum has been shown to protect against inflammation and PF induced by PDFs, resulting in an increase in the ratio of Tregs to Th17 cells (162). Another study suggests that T lymphocytes stimulates IL-17 expression by releasing calpains to regulate TLR2 (163).

Recent evidence also suggests that the IL-17A-induced inflammatory response may contribute to peritoneal injury in both experimental models induced by PDFs and in patients undergoing PD (94, 146, 160, 164). Studies in mice and human peritoneal biopsies have also demonstrated an overexpression of IL-17 (151). It is possible that endogenous factors in the peritoneum, such as AGEs, might regulate IL-17 levels by promoting pro-inflammatory cytokines IL-6 and TGF-β (165, 166). Binding IL-17A to its receptor in mesothelial cells has the ability to trigger proinflammatory responses. Studies using cultured HPMCs have shown that IL-17A can activate the NF-κB pathway and the release of downstream cytokines, such as CXCL1 (167, 168). Additionally, IL-17A has been found to stimulate peritoneal cells, resulting in the upregulation of proinflammatory cytokines like IL-1β, IL-6 and MCP-1, which contribute to the persistence of inflammation (48, 169). Resident γδ T cells in peritoneum produce IL-17, which in turn contributes to fibrosis and ultrafiltration failure (94). Conversely, studies have shown that intraperitoneal injections of a neutralizing IL-17A antibody can prevent peritoneal changes and reduced PF in mice exposed to PDFs (94).

**Figure 5:**
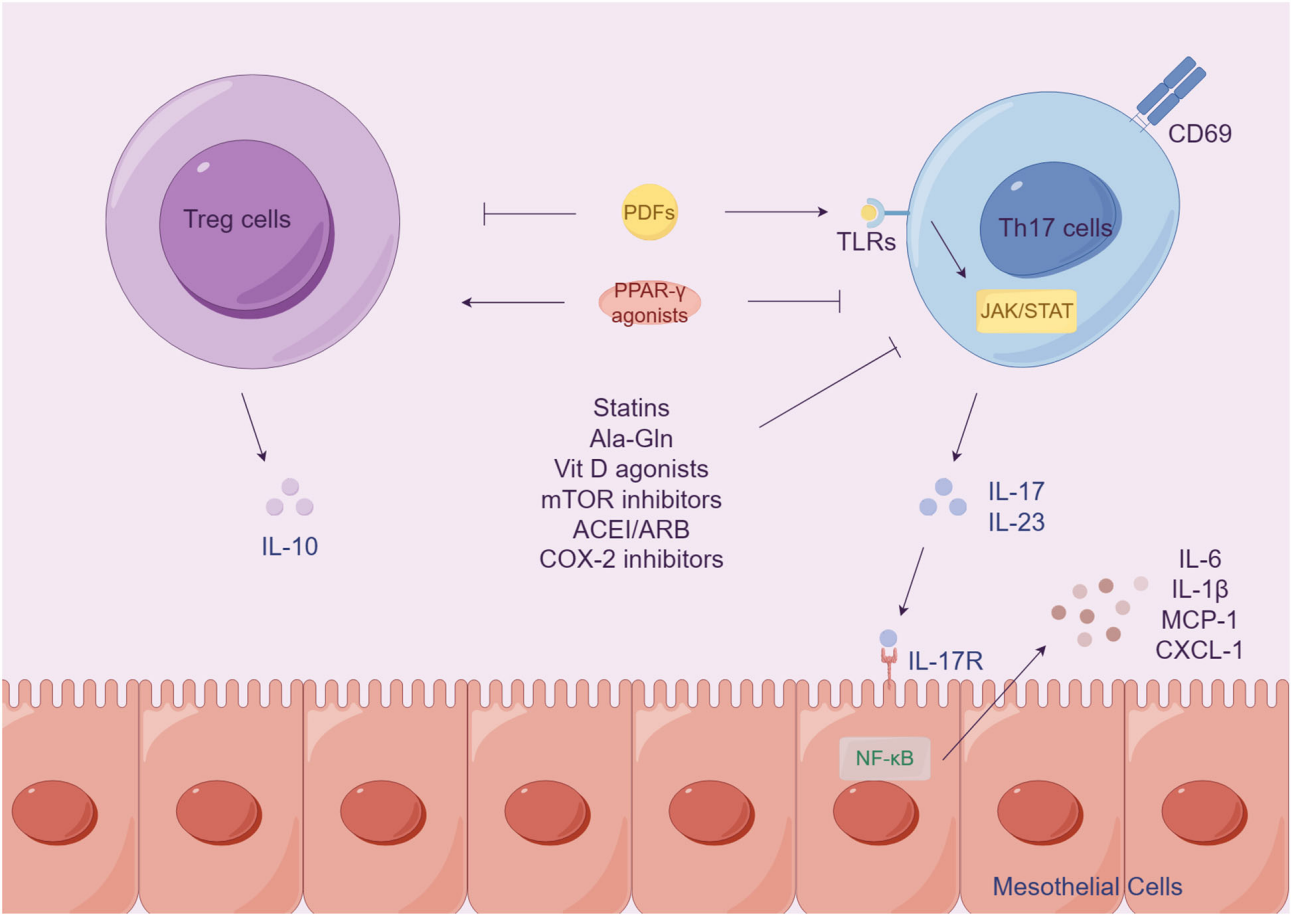
Th17/Treg axis contributes to peritoneal damage induced by PDFs. Prolong exposure to dialysis fluids results in an imbalance of Th17/Treg cells, with an up-regulation of Th17 cells and down-regulation of Treg cells, ultimately leading to peritoneal damage. IL-17A released by activated Th17 cells stimulate peritoneal cells, leading to the upregulation of proinflammatory cytokines. Nevertheless, therapeutic approaches targeting the T17/Treg-axis may be beneficial to reduce peritoneal injury.

Various therapeutic approaches aimed at preventing peritoneal damage by targeting the Th17/Treg axis have been investigated (**Figure 5**). In PD patients, treatment with rosiglitazone has been shown to lower levels of IL-17 and IL-23 and increase levels of FoxP3+Treg activity (64). Paricalcitol, the vitamin D activator, also inhibits IL-17 production and slows the progression of PF (170). The dipeptide alanyl-glutamine has also been found to ameliorate PF and attenuate IL-17-dependent pathways during PD (171). Additionally, it has been discovered that the use of conventional PDFs triggers the activation of the Th17 immune response in the peritoneum, while the use of biocompatible PDFs does not (146). Moreover, potential therapeutic options include statins, mammalian target of rapamycin inhibitors, cyclooxygenase-2 inhibitors, and angiotensin converting enzyme inhibitors, as they have shown the ability to modulate the Th17/IL-17A response in the injured peritoneum (172).

Besides, the presence of AGEs has been hypothesized to be responsible for the proliferation of CD8+ lymphocytes (153, 173). It is believed that repeated exposure to the dialysate can lead to the recruitment and transformation of CD8+ naïve T cells into CD8+ effector memory cells (174).

#### 2.2.3 Mast cells: the controversial effects

The role and specific mechanism of mast cells in PD remain a topic of controversy. Several studies have reported an elevated presence of mast ocytes in samples obtained from PD patients (175). Additionally, both chronic renal failure rat models and PD rat models have shown a significant increase in mast cell numbers in the peritoneum (4, 176, 177). However, a preliminary investigation observed a decrease in mast cell quantification in peritoneal biopsies of patients (178). The influence of mast cells on inflammatory and fibrotic processes is depends on the timing, intensity, or nature of the damaging stimulus (179, 180). Mast cells that reside in the peritoneum have the potential to modulate the functioning of mesothelial cells through the release of their mediators, primarily histamine. This modulation occurs partly through calcium-dependent pathways and can affect the functionality of the peritoneum in the context of PD (181). In addition, mast cells play a role in the remodeling of omental tissue caused by PDFs, leading to the migration of peritoneal cells and the formation of adhesion (182).

#### 2.2.4 Other immune cells: an almost blank field

Both hemodialysis and PD patients experience a significant decrease in the population of natural killer cells, which are essential components of the innate immune system (183). This highlights a strong link between ESRD and immune activation, as well as immune deficiency. Notably, another study indicates that the count of natural killer cells does not show any correlation with the duration of CAPD (184).

As previously discussed, TLRs, specifically TLR2 and TLR4, play a crucial role in the development of sterile peritoneal inflammation due to prolonged exposure to PDFs. However, inhibiting TLR2 has been shown to reduce the number of leukocytes in the peritoneum, particularly the infiltrated neutrophils (185). Furthermore, research suggests that the migration of neutrophil during inflammation is influenced by the compatibility of PDFs (101). Despite the documented biocompatibility of currently used catheters, there is extensive evidence of increased eosinophil counts in both peripheral blood and peritoneal fluid following catheter replacement. This response can range from a mild increase in asymptomatic individuals to a severe elevation that can ultimately lead to eosinophilic peritonitis (186–191). Eosinophilic peritonitis is characterized by aseptic inflammation, manifested as cloudy peritoneal dialysis effluent, mild clinical symptoms, negative dialysate culture, and lack of response to antibiotic treatment (191). Tissue invasion, resulting from infection and/or nonspecific stimulation of the PDFs, triggers the release of danger signals that activate eosinophils. This activation initiates the pathogenesis of the innate immune system, aimed at protecting the body but ultimately inducing tissue fibrosis (192).

## 3 Conclusion and prospect

The cellular and molecular mechanisms mentioned above emphasize the complex nature of the pathophysiological response observed in the peritoneum. The interaction between stromal resident cells and immune cells is mutually influential. Resident cells play an active role in influencing the recruitment, survival, and differentiation of immune cells, On the other hand, immune cells regulate the expression of proinflammatory factors and cytokines, which can cause damage to peritoneal resident cells.

Despite notable progress in PD in recent years, the issue of sterile inflammation remains a significant complication that can result in technique failure and unfavorable clinical outcomes. The repeated exposure to bioincompatible PDFs continues to be the primary cause of sterile inflammation. To overcome this challenge, future research efforts should focus on enhancing the biocompatibility of PDFs to improve peritoneal viability and extend the duration of PD therapy. Currently, no PDFs meet all the ideal solution requirements, which include efficient ultrafiltration, long-term preservation of the peritoneal membrane, and correction of nutritional and metabolic abnormalities.

However, the use of novel PDFs in conjunction has the potential to achieve these objectives. Initial findings from clinical investigations suggest that these biocompatible PDFs can provide comparable effectiveness to conventional regimens while also offering superior preservation of mesothelial cell mass (193). Furthermore, there are evidences to suggest that biocompatible PDFs can induce peritoneal inflammation and angiogenesis in children undergoing PD treatment (194, 195). The activation of peritoneal cells, mediators, and pathways can result in long-lasting functional and structural changes in the peritoneal membrane during long-term PD therapy. However, certain modifications may be reversible through the implementation of peritoneal rest (4, 196). The possibility of remesothelialization or healing depends on allowing the peritoneum to rest. During this resting period, pluripotent cells migrate to the surface and differentiate into fully developed mesothelial cells (197–199). Normal stem cells also play a crucial in tissue regeneration after injury, either by being mobilized from the bone marrow or already present in damaged tissues (147, 200). Furthermore, gene therapy can be used to modify the peritoneal membrane by targeting intervention to control inflammation, fibrosis and angiogenesis. These novel approaches show promising potential in preserving the integrity of the peritoneal membrane (201, 202). However, their clinical efficacy is still to be determined. Additionally, there is ongoing investigation into the potential clinical significance of using catheters infused with antimicrobial agents to prevent infections associated with PD (203). Consequently, we anticipate the potential use of catheters impregnated with anti-inflammatory medications as a therapeutic approach to reduce catheter-induced alterations and offer benefits to patients undergoing PD. However, it is important to conduct additional testing to ensure the safety and effectiveness of these PD tubes.

These events can potentially trigger both acute or chronic inflammation, resulting in damage to the peritoneal membrane and a gradual deterioration of its functioning. The role of mesothelial cells and other recruited cells is essential in initiating sterile inflammation and the subsequent decline of the peritoneal membrane. The inflammatory response is intricately intertwined with a complex network of extracellular signals produced by various types of cells residing or circulating within the peritoneal membrane. Here, we offer a thorough depiction of the key extracellular factors and cellular components that contribute to the communication between the immune system and peritoneal stromal cells. The exploration of the intricate cellular and molecular mechanisms involved in the sterile inflammation of the peritoneal membrane carries substantial significant in both fundamental research and clinical practices. This comprehension contributes to the development of therapeutic approaches focused on alleviating deterioration and reinstating the homeostasis of the peritoneal membrane.

## Author contributions

HS: Writing – original draft. RZ: Writing – original draft. JS: Writing – review & editing. XC: Writing – review & editing. HY: Writing – review & editing. NA: Writing – review & editing. CY: Writing – review & editing. JT: Writing – review & editing. HL: Writing – review & editing. CY: Writing – review & editing.
